# Hepatocellular Carcinoma More Than 3 cm in Diameter: A Systematic Review of Transcatheter Arterial Chemoembolization Plus Percutaneous Ethanol Injection versus Transcatheter Arterial Chemoembolization Alone

**DOI:** 10.1155/2013/526024

**Published:** 2013-07-01

**Authors:** Shiying Wang, Liping Zhuang, Zhiqiang Meng

**Affiliations:** ^1^Department of Integrative Oncology, Fudan University Shanghai Cancer Center, 270 Dong An Road, Shanghai 200032, China; ^2^Department of Oncology, Shanghai Medical College, Fudan University, 130 Dong An Road, Shanghai 200032, China

## Abstract

*Objective*. To identify the efficiency and safety of transcatheter arterial chemoembolization (TACE) combined with percutaneous ethanol (PEI) for patients with hepatocellular carcinoma (HCC) more than 3 cm in diameter in comparison with those of transcatheter arterial chemoembolization monotherapy. *Methods*. All databases were searched up to February 22, 2013. The literature retrieval was conducted through Pubmed, Web of Science, and Cochrane Library. We also searched Chinese databases, including Chinese National Knowledge Infrastructure (CNKI), Chinese Biology Medicine (CBM), Wanfang database, and VIP Database for Chinese Technical Periodicals without language limitations. *Results*. Based on the criteria, we found 12 RCTs including 825 patients. Our results showed that TACE combined with PEI therapy compared with TACE monotherapy improved overall survival and tumor response. *Conclusion*. The combination of TACE and PEI compared with TACE monotherapy improved overall survival rates and tumor response of patients with large HCC. Besides, larger and more methodologically rigorous clinical trials are needed to confirm this outcome.

## 1. Introduction

Transcatheter arterial chemoembolization (TACE) has been widely used in the treatment of hepatocellular carcinoma (HCC), so does percutaneous ethanol injection (PEI). However, the effectiveness of PEI is limited to small lesions less than 3 cm in diameter [1]. The possibility of profitably combination of intra arterial therapies with ethanol injection in large HCC was first suggested by Tanaka et al. [2]. However, previous studies assessing the effectiveness of TACE combined with PEI versus TACE alone reported conflicting results in large HCC [3–6]. Becker et al. [4] showed no significant difference in the overall survival among large HCC patients under combination therapy or monotherapy, whereas several studies demonstrated that the combination of TACE and PEI was more effective than TACE monotherapy for large tumors [3, 5, 6]. In 2011, a meta-analysis assessed the effectiveness of TACE and PEI; however, the tumor size in most studies included in this review was unclear [7]. So whether combination of TACE and PEI was more effective than TACE alone in large HCC is still unknown. To determine the effectiveness of the combination of TACE and PEI in HCC more than 3 cm in diameter, we performed this meta-analysis using the grading of recommendations, assessment, development, and evaluation (GRADE) system [8, 9].

## 2. Methods

### 2.1. Search Strategy

All databases were searched up to February 22, 2013. We searched PubMed, Web of Science, and Cochrane Library involving the following terms: (“carcinoma, hepatocellular” (MeSH)) AND (“embolization” (MeSH)) AND (“ethanol injection”) AND (“randomized controlled trail” (Publication Type)). We also searched Chinese databases, including Chinese National Knowledge Infrastructure (CNKI), Chinese Biology Medicine (CBM), Wanfang database, and VIP Database for Chinese Technical Periodicals. Chinese language database was retrieved with similar search strategy. The literature was searched by 2 authors (S.-Y. Wang and L.-P. Zhuang), and any inconsistencies were discussed with a third author (Z.-Q. Meng).

### 2.2. Criteria for Inclusion

Studies which complied with the following criteria were included: (1) type of studies: randomized controlled clinical trial; (2) participants: patients with large (>3 cm) HCC were diagnosed cytologically or pathologically, or diagnosed by CT; all patients were untreated and no evidence for extrahepatic metastases; (3) Type of intervention: studies compared TACE combined with or without PEI; (4) Type of outcome measurements: overall survival rate, and tumor response were the main outcome measurements.

### 2.3. Criteria for Exclusion

Trials were excluded if they did not meet the criteria previously and including the following criteria: (1) involved animal studies or in vitro studies; (2) did not represent primary research (review articles; letter to the editor, etc.); (3) represented duplicate publications of other studies.

### 2.4. Outcome Measurements

Outcome measurements of these trials comprised overall survival and tumor response. Overall survival measures included 1-, 2- and 3-year survival rate. In accordance with the World Health Organization (WHO) standard for evaluating therapeutic efficacy on solid tumors (11), tumor response was defined as follows: complete response (CR) refers to complete disappearance of the lesion on CT and/or MRI images; partial response (PR) refers to lesion decreased ≥50%; stable disease (SD) refers to lesion decreased less than 50% or increased less than 25%; and the size of any lesion increased more than 25% or the appearance of new lesions is considered as progressive disease (PD).

### 2.5. Data Extraction

Two reviewers (S.-Y. Wang and L.-P. Zhuang) independently selected the trials and conducted data extraction. Discrepancies were settled through the involvement of a third reviewer (Z.-Q. Meng) in consensus conferences. If the required information is unavailable in the original published articles, we obtained additional information in correspondence with the authors. The following information was extracted from each report: authors, time of publication, numbers of patients in TACE combined with PEI group and TACE alone group, age, Child-Pugh class, tumor size, and number of tumors. For studies using other agents as the third arm, only the two arms using TACE with/without PEI will be included for analysis.

### 2.6. Quality Assessment

The methodological quality of each randomized control trial was assessed according to The Cochrane Collaboration's tool described in Handbook version 5.1.0 [19]. Two authors (S.-Y. Wang and L.-P. Zhuang) assessed the quality independently, and inconsistency was discussed with a third review author (Z.-Q. Meng), who acted as arbiter.

### 2.7. Statistical Method

The meta-analysis was carried out according to the Cochrane Reviewer's Handbook recommended by The Cochrane Collaboration. All *P* values were two-sided, and *P* < 0.05 was regarded as statistically significant. For dichotomous variables, relative risk (RR) was calculated with 95% confidence interval (CI). Statistical heterogeneity was performed using the chi-square test (*P* < 0.10 was considered representative of significant statistical heterogeneity). Meta-analysis of studies with an acceptable level of heterogeneity (*P* > 0.05, or *P* < 0.05 but *I* ^2^ ≤ 50) was conducted using a fixed-effects model. A random-effects model was used for studies where significant heterogeneity was found (*P* ≤ 0.05, but *I* ^2^ > 50%). Data from RCTs meeting inclusion criteria was analyzed with Review Manager (version 5.1 for Windows; The Cochrane Collaboration, Oxford, UK).

## 3. Results

### 3.1. Search Results

**Figure 1:**
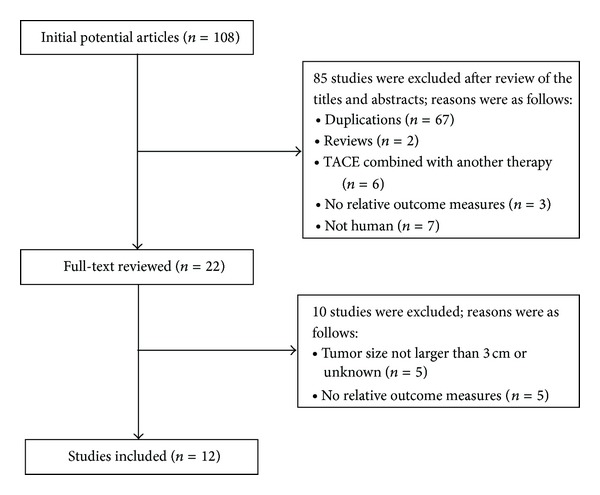
Flowchart of the study selection process.

Figure 1 describes the procedure for selecting eligible trials. Our search yielded 108 citations. After review of the titles and abstracts, we excluded 86 studies. Ten studies were excluded after full-text review. Eventually, Twelve studies were included.

### 3.2. Study Characteristics and Quality Assessment

12 RCTs investigating the therapeutic effect of TACE and PEI were included in this meta-analysis [3, 5, 6, 10–18]. The total number of patients was 825, with 422 patients in TACE plus PEI group and 403 patients in TACE group. The PEI procedure was conducted in 1 to 4 weeks after TACE in all trails. Few studies reported that total number of TACE and PEI procedures in each group. Bartolozzi et al. [5] reported that TACE was performed once and PEI was performed from 6 to 16 times on (mean ± standard deviation, 7.8 ± 2.6) each patient in TACE plus PEI group, while TACE was performed twice in TACE alone group. Xu et al. conducted TACE 3.4 times and PEI 4.7 times in average in TACE plus PEI group and performed TACE 3.7 times in average in TACE alone group. The volume of ethanol injected was reported only in two trails which was 31.4 mL and 65.4 ± 43.7 mL according to tumor size, respectively [5, 6]. Characteristics of studies were listed in Table 1.

**Figure 2:**
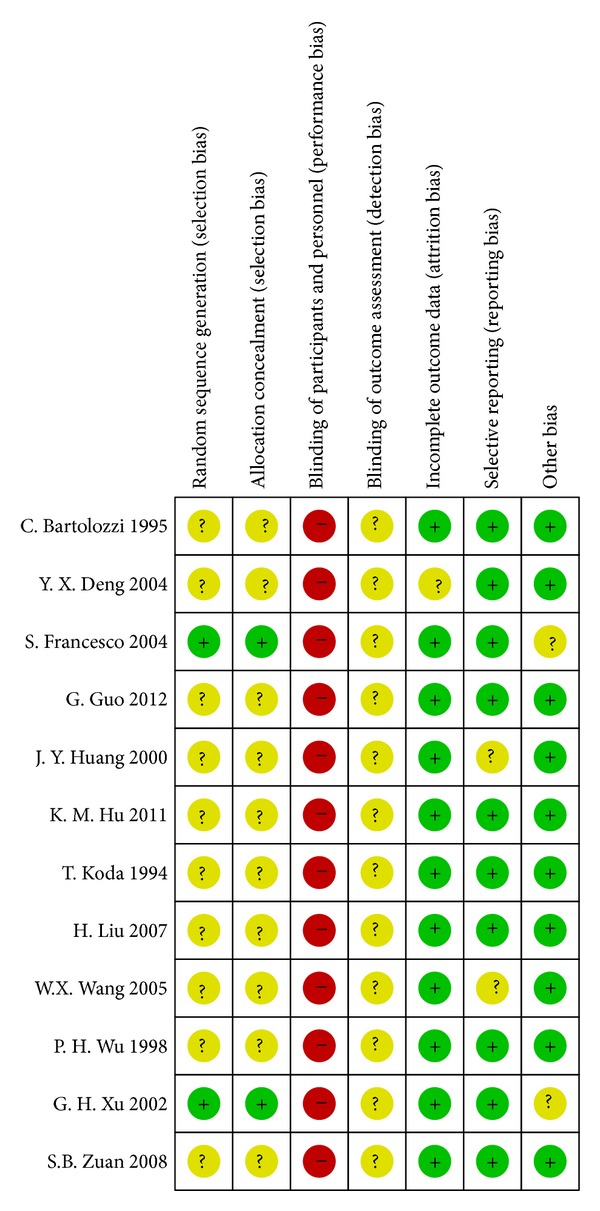
Risk of bias summary: review authors' judgments about each risk of bias item for each included study.

**Figure 3:**
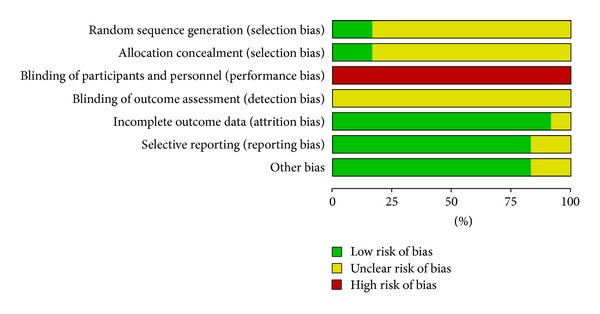
Risk of bias graph: review authors' judgments about each risk of bias item presented as percentages across all included studies.

All trials stated “randomization,” but few reported the generation of a random allocation sequence in detail. The double-blinded approach was unable to be displayed in all trials because of the intrinsic nature of the interventional treatments. None of trials mentioned the blinding of outcome assessment, so the detection bias was unclear to us. The quality assessment was performed using The Cochrane Collaboration's tool, and the outcome was shown in Figures 2 and 3.

### 3.3. Meta-Analysis

#### 3.3.1. One-Year Survival Rate

**Figure 4:**
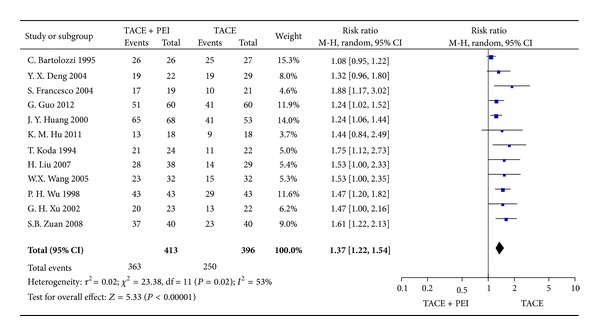
1-year survival rates of TACE+PEI versus TACE (random-effects model).

We identified 12 trials (809 patients) with outcome measurement of 1-year survival rates. Meta-analysis indicated a significant improvement in the 1-year survival favoring TACE combined with PEI over TACE alone (RR = 1.37, 95% CI 1.22–1.54, *P* < 0.00001) (Figure 4).

#### 3.3.2. Two-Year Survival Rate

**Figure 5:**
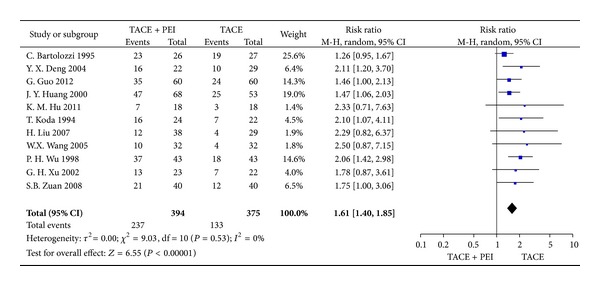
2-year survival rates of TACE+PEI versus TACE (random-effects model).

Eleven trials (769 patients) assessed the outcome measurements of 2-year survival. There is no significant heterogeneity among studies (Chi^2^ = 9.03, *P* = 0.53). Meta-analysis showed that TACE combined with PEI group improved the survival at 2 years compared with TACE group (RR = 1.61, 95% CI 1.40–1.85, *P* < 0.00001) (Figure 5).

#### 3.3.3. Three-Year Survival Rate

**Figure 6:**
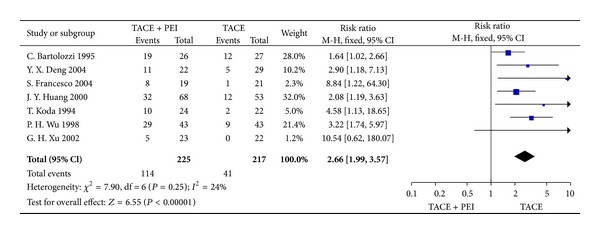
3-year survival rates of TACE+PEI versus TACE (fixed-effects model).

Seven studies (442 patients) were selected for the meta-analysis. The heterogeneities showed the results had no significant difference among the pooled individual studies (Chi^2^ = 7.90, *P* = 0.25). Three-year survival rate assessment in the treatment of large HCC was significantly in favor of TACE combined with PEI group than TACE group (RR = 2.66, 95% CI 1.99–3.57, *P* < 0.00001) (Figure 6).

### 3.4. Tumor Response

**Figure 7:**
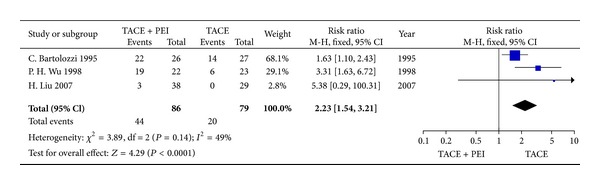
CR rates of TACE+PEI versus TACE (fixed-effects model).

**Figure 8:**
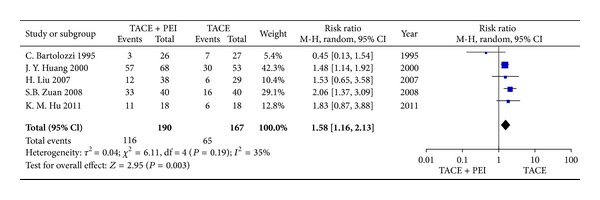
CR+PR rates of TACE+PEI versus TACE (random-effects model).

Three studies reported a CR outcome measurement and five studies reported a CR+PR outcome measurement. Seven studies reported a PR outcome measurement and demonstrated a higher PR rate favoring TACE combined with PEI group compared with TACE alone group, except one study [5]. Meta-analysis indicated that TACE combined with PEI group significantly improved tumor response (RR_CR_ = 2.23, 95% CI 1.54–3.21, *P* < 0.0001;  RR_CR+PR_=1.84, 95% CI 1.17–2.92, *P* < 0.05) (Figures 7 and 8).

### 3.5. GRADE Evidence Profile

Quality of evidence evaluated by GRADE system was listed in Table 2. Although selection bias of most trials included in our systematic review was unclear, the overall quality of evidence was graded to be moderate to high quality. So the outcomes of our meta-analysis were likely to be reliable, but still needed to be further confirmed. 

## 4. Discussion

TACE is one of the most common interventional therapies in HCC patients. But because HCC often has intracapsular or extracapsular invasion and viable tumor cells remain after TACE, it is difficult to achieve complete necrosis of the target tumor by TACE monotherapy [20]. Therefore TACE needs to be repeated to achieve better result; however, the modality still does not yield sufficient control of the growth of HCC when it is used alone [21]. Repeated TACE may also cause liver function to worsen because of the damaging of nontumorous liver parenchyma [22]. PEI is also widely used in the treatment of HCC. However, the effectiveness of PEI is limited to small lesions less than 3 cm in diameter [1]. In large hepatocellular lesions, ethanol diffusion within the tumor was incomplete and could be washed out by the high vascularity of HCC.

The possibility of profitably combining intraarterial therapies with ethanol injection in the treatment of HCC was first suggested by Tanaka et al. [2]. Because prior transcatheter embolization makes the tumor parenchyma necrotic and enables the administration of a large volume of ethanol, filling the entire tumor with ethanol under high pressure and resulting in complete necrosis of even large lesions [6], The enhanced ethanol diffusion secondary to necrotic changes produced by TACE and washout of the injected ethanol is more difficult in the timorous area [2]. However, previous studies assessing the effectiveness of TACE combined with PEI versus TACE alone reported conflicting results in large HCC [3–6]. Becker et al. [4] showed no significant difference in the overall survival among large HCC patients under combination therapy or monotherapy, whereas several studies demonstrated that the combination of TACE and PEI was more effective than TACE monotherapy for large tumors [3, 5, 6].

We performed this meta-analysis to provide the most comprehensive assessment of the combination of TACE and PEI compared with TACE alone for large HCC. Finally, 12 studies were included into this meta-analysis. Our meta-analysis showed that the combination of TACE and PEI was associated with higher survival rates (RR_1-year_ = 1.37, 95% CI 1.22–1.54, *P* < 0.00001; RR_2-year_ = 1.61, 95% CI 1.40–1.85, *P* < 0.00001; RR_3-year_ = 2.66, 95% CI 1.99–3.57, *P* < 0.00001) and higher tumor response (RR_CR_ = 2.23, 95% CI 1.54–3.21, *P* < 0.0001; RR_CR+PR_ = 1.84, 95% CI 1.17–2.92, *P* < 0.05). The outcomes from our review suggested that the efficacy of the combination of TACE and PEI was significantly better than that of TACE alone in large HCC. However, there was only one high quality outcome assessed by GRADE profile and the quality of other outcomes was moderate. Thus, the results mentioned previously still need further confirmation by high quality randomized controlled trials.

The risk of bias in our meta-analysis was assessed by Cochrane Collaboration's tool and the outcomes showed that there was unclear risk of selection bias in the 12 trials. GRADE is an emerging system of rating quality of evidence and grading strength of recommendations in systematic reviews, health technology assessments (HTAs), and clinical practice guidelines addressing alternative management options [9]. We strengthened the evidence in our meta-analysis by using GRADE system. Quality of evidence evaluated by GRADE system was listed in Table 2. Although selection bias of most trials included in our systematic review was unclear, the overall quality of evidence was graded to be moderate to high quality. So the outcomes of our meta-analysis were likely to be reliable, but still needed to be further confirmed.

There are still some limitations in this meta-analysis. Firstly, randomization and allocation concealment were not clearly described in most of the included trials, which may result in the emergence of selection bias and overestimation of the efficacy of the treatment group. Thus, most trials included in this study were of generally unclear selection bias; it may put the results of meta-analysis in risk. Secondly, because of the intrinsic nature of the interventional treatments, TACE and PEI interventional treatments are hard to implement in a blinded way. Thirdly, publication bias may exist in the present study. Most of the findings presented in the included studies are positive results. Some negative results may be unreported and therefore are not included in the review. Finally, most of the trials included in the meta-analysis without adequate description of demography and methodology such as duration of cancer and intention-to-treat analyses. Larger and more methodologically rigorous clinical trials are needed to confirm the findings in the meta-analysis.

The included studies were inadequate, so we suggest that future researches should be adequate description of randomization sequence generation, allocation concealment, and randomization implementation. The enrolled patients should be included in the analysis of outcomes with an intention-to-treat analysis to determine the overall cost utility of a treatment. Therefore, more rigorously designed, multicenter, randomized, controlled trials are still required.

In conclusion, the combination of TACE and PEI compared with TACE monotherapy improved overall survival rates and tumor response of patients with large HCC. Besides, larger and more methodologically rigorous clinical trials are needed to confirm this outcome.

## Figures and Tables

**Table 1:** Characteristics of RCTs included in the meta-analysis.

Study ID	Group	*N*	Age	Child-Pugh (A/B/C)	Tumor size (mm)	Number of tumors (1/≥2)
Bartolozzi et al. 1995 [5]	TACE + PEI	26	65.3 ± 6.2	14/12/0	48 ± 14	18/8
	TACE	27	66.1 ± 4.9	11/16/0	51 ± 14	14/13
Deng 2004 [10]	TACE + PEI	22	54.1 ± 10.5	13/6/3	68 ± 17	—
	TACE	29	54.5 ± 11.2	16/8/5	68 ± 19	—
Ferrari et al. 2004 [3]	TACE + PEI	19	—	16/3/0	54 ± 16	18/1
	TACE	21	—	10/11/0	55 ± 26	20/1
Guo et al. 2012 [11]	TACE + PEI	60	55 ± 11	—	64 ± 24	—
	TACE	60	54 ± 12	—	65 ± 18	—
Huang et al. 2000 [12]	TACE + PEI	68	45.2	—	≥40	—
	TACE	53	45.2	—	≥40	—
Hu et al. 2011 [13]	TACE + PEI	18	46	15/3/0	78	—
	TACE	18	67	16/2/0	81	—
Kato et al. 1994 [6]	TACE + PEI	24	61.3	19/5/0	65	—
	TACE	22	64.5	17/5/0	71	—
Liu et al. 2007 [14]	TACE + PEI	38	47	22/7/0	>30	25/13
	TACE	29	49	30/8/0	>30	18/11
Wang et al. 2005 [15]	TACE + PEI	32	—	—	>50	—
	TACE	32	—	—	>50	—
Peihong et al. 1998 [16]	TACE + PEI	52	55 ± 18	40/8/2	52 ± 23	—
	TACE	50	55 ± 16	40/9/3	52 ± 21	—
Xu et al. 2002 [17]	TACE + PEI	23	—	—	>50	—
	TACE	22	—	—	>50	—
Zan et al. 2008 [18]	TACE + PEI	40	—	—	>30	—
	TACE	40	—	—	>30	—

**Table 2:** Assessment of quality using the GRADE system.

Quality assessment							Quality
Outcomes	Number of studies	Risk of bias	Inconsistency	Indirectness	Imprecision	Other considerations	
1-year survival rate	12	Serious^1^	No serious inconsistency	No serious indirectness	No serious imprecision	None	⨁⨁⨁O MODERATE
2-year survival rate	11	Serious^2^	No serious inconsistency	No serious indirectness	No serious imprecision	None	⨁⨁⨁O MODERATE
3-year survival rate	7	Serious^3^	No serious inconsistency	No serious indirectness	No serious imprecision	Reduced effect for RR ≫ 1 or RR ≪ 1^3,4^	⨁⨁⨁⨁ HIGH
CR	3	Serious^5^	No serious inconsistency	No serious indirectness	No serious imprecision	None	⨁⨁⨁O MODERATE
CR + PR	5	Serious^6^	No serious inconsistency	No serious indirectness	No serious imprecision	None	⨁⨁⨁O MODERATE

^1^All trials stated randomization, but random sequence generation was unclear in 10 of 12 included trials.

^2^All trials stated randomization, but random sequence generation was unclear in 9 of 11 included trials.

^3^All trials stated randomization, but random sequence generation was unclear in 5 of 7 included trials.

^4^RR 2.66 ≫ 1 (1.99, 3.57).

^5^All trails stated randomization, but random sequence generation was unclear in 3 included trials.

^6^All trials stated randomization, but random sequence was unclear in 5 included trials.
